# Urban Household Carbon Emission and Contributing Factors in the Yangtze River Delta, China

**DOI:** 10.1371/journal.pone.0121604

**Published:** 2015-04-17

**Authors:** Xibao Xu, Yan Tan, Shuang Chen, Guishan Yang, Weizhong Su

**Affiliations:** 1 Key Laboratory of Watershed Geographic Sciences, Nanjing Institute of Geography and Limnology, Chinese Academy of Sciences, Nanjing, China; 2 Department of Geography, Environment and Population, The University of Adelaide, Adelaide, Australia; Shandong University, CHINA

## Abstract

Carbon reduction at the household level is an integral part of carbon mitigation. This study analyses the characteristics, effects, contributing factors and policies for urban household carbon emissions in the Yangtze River Delta of China. Primary data was collected through structured questionnaire surveys in three cities in the region – Nanjing, Ningbo, and Changzhou in 2011. The survey data was first used to estimate the magnitude of household carbon emissions in different urban contexts. It then examined how, and to what extent, each set of demographic, economic, behavioral/cognitive and spatial factors influence carbon emissions at the household level. The average of urban household carbon emissions in the region was estimated to be 5.96 tonnes CO_2_ in 2010. Energy consumption, daily commuting, garbage disposal and long-distance travel accounted for 51.2%, 21.3%, 16.0% and 11.5% of the total emission, respectively. Regulating rapidly growing car-holdings of urban households, stabilizing population growth, and transiting residents’ low-carbon awareness to household behavior in energy saving and other spheres of consumption in the context of rapid population aging and the growing middle income class are suggested as critical measures for carbon mitigation among urban households in the Yangtze River Delta.

## Introduction

The Fifth Assessment Report of the IPCC asserts that anthropogenic greenhouse gas emission has warmed the Earth’s climate and consequently climate change poses an increasing impact on natural and human systems [1]. As the world’s largest carbon emitter, China accounted for 29% of global total emissions in 2012 and 80% of the world’s increase in carbon emissions since 2008 [2]. The magnitude of CO_2_ emissions is expected to continue to grow because of sustained industrialization and urbanization of China’s economy. To combat global climate change and sustainable development, the Chinese central government intends to achieve the peaking of carbon dioxide emission around 2030 [3]. Household consumption, capital investment and growth in exports are the three main forces driving CO_2_ emissions in China from 1980 to 2030, and more than a third (38%) of the net addition (2,277 MMT) of CO_2_ emissions in China from 1981 to 2002 was generated by urban households [4]. Household consumption has been a major contributor to total carbon emissions on the national and regional scales [4–6]. CO_2_ emissions coming directly from household consumption accounted for 29.7% of total emissions in China in 2010 [7]. Strikingly, per capita annual carbon emission of urban residents is 2.6 times that of rural residents (1.21 tonnes per head) [8]. Urban households weigh heavily in CO_2_ emissions and thus must be taken into account in policy making to build an energy-saving and low-carbon economy in China and to realize its commitment to the world’s carbon mitigation obligations.

There has been a substantial amount of research completed on the methods for estimating the quantity and for analyzing the characteristics of and mechanisms for carbon emissions at the *macro* (from global to local) scale [5, 9, 10]. At the *micro* (household) level, international studies of household carbon emissions have addressed the impact of demographic and social-economic factors and changing consumption patterns [11, 12]. Significant factors influencing carbon emissions are identified to be: age structure [13, 14], gender [15, 16], household size [17], housing area [11], household income [18, 19], consumption behavior [11, 12, 20], food consumption [21, 22], and urban spatial structure [23]. Some sophisticated methodology and methods for estimating carbon emissions have been established and applied widely, including the IPAT model [24], STIRPAT model [10], Consumer Lifecycle Approach (CLA) [9, 25], Input-output model [26] and hybrid-EIO-LCA method [27]. However, some important factors, such as the effect of disparity in incomes among households, a growing middle income class and resultant changes in their consumption behavior, and diverse energy pricing and energy-saving policies implemented in different urban contexts are still understudied. This is particularly the case as it relates to urban areas and urban households in China. A lack of clarity of how these important factors influence carbon emissions could have a negative impact on mitigation policies including a carbon tax, global negotiations about future emission targets and the differentiated responsibilities between stakeholders at all levels [18].

This study addresses two issues: firstly, what are the current magnitude and major sources of carbon emissions at the urban household level? Secondly, in what ways and to what extent do demographic, economic, behavioral/cognitive and spatial factors impact on carbon emissions of urban households? This paper seeks to address these issues by focusing on the Yangtze River Delta (YRD) and by employing unique primary data collected in three cities (Nanjing, Ningbo, Changzhou) of the YRD in August-October 2011 for analysis. The region contains the nation’s largest urban cluster—one that comprises 16 major cities. The YRD has been undergoing fast demographic and social-economic transition, characterized by significant change in economic structure, dramatic population growth caused by migration and urbanization, and enhanced household wealth and urban lifestyles since 1990 [28]. It provides a particularly salient place to study these issues. Recent research into carbon emissions in the YRD highlights the effect of macro factors, especially economic and industrial development in the region [29, 30]. However, there is little research disentangling the complex nexus between carbon emission pattern, mechanisms and mitigation policy in the region from a *micro* (household) perspective [20, 31, 32]. These challenges make it urgent to identify the key factors influencing urban household carbon emissions and to formulate effective energy-saving and carbon mitigation polices in this region as it closely relates to the success or failure of China’s 12^th^ Five-Year Energy-Saving Plan (2011–2015) set out by the State Council. In the Plan, the YRD was assigned the largest reduction task in the country: reducing total energy consumption by 18% and CO_2_ emissions per unit of GDP by 19% by 2015 based on the 2005 levels, which are both two percentage points higher than the national targets, respectively. This study is therefore fundamental in understanding the pathway towards carbon mitigation in the YRD or even in China.

Carbon emissions generated by domestic energy consumption (including electricity, water, natural gas, and liquefied petroleum gas—LPG), individual transportation (daily commuting and long-distance travel) and garbage disposal were particularly addressed in this study. The estimation of total household carbon emissions and their composition is built upon the 2006 Intergovernmental Panel on Climate Change Guidelines for National Greenhouse Gas Inventories [33]. Descriptive statistical analysis and ordinary least squares (OLS) regression are used for analysis of the survey data. Practical countermeasures to reduce urban household carbon emissions are suggested, based on the findings of the study.

## Materials and Methods

### Study Area

**Fig 1:**
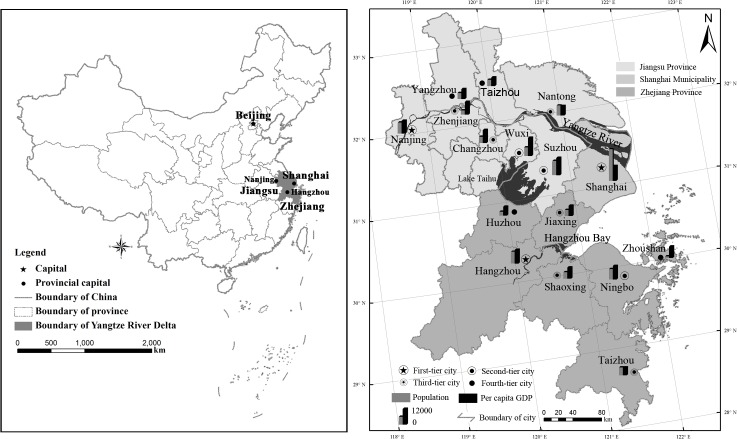
Population and per capita GDP across 16 major cities in the Yangtze River Delta, 2010. This is the Fig. 1 legend. Created with the ArcGIS 10.0 software. Notes: Figures shown in bars of the map were calculated based on 2010 China Census data, and measured in 1,000 persons for population and Chinese yuan for per capita GDP (USD 1 = RMB 6.77 yuan, the annual average exchange rate in 2010). First-tier cities include three provincial capitals (Shanghai, Hangzhou, Nanjing), each with a population of 5 million or more. Second-tier cities are large-scale cities with a population of 3–5 million, which includes three cities (Suzhou, Wuxi, Ningbo). Third-tier cities are medium-scale cities with a population of 1–3 million, including Taizhou (Zhejiang), Shaoxing, Nantong, Changzhou, Jiaxing, and Zhenjiang. Fourth-tier cities are the relatively small-scale cities of Yangzhou, Huzhou, Zhoushan, and Taizhou (Jiangsu).

The YRD (located within E118º20′-122º46′, N28º2′-33º25′) is one of the most populous and developed regions of China and one of six megalopolitan regions in the world. The 16 major cities in the region can be classified into four tiers, grouped in terms of their population size, economic output, and roles in the national and regional economy [34] (Fig. 1). The three cities under study—Nanjing (with a population of 6.32 million), Ningbo (2.02 million), Changzhou (1.62 million)—are located at the First-, Second- and Third-tier panel, respectively. The delta area has the largest regional economic capacity in China, and its gross regional production (GRP) accounted for 17.6% (or 39,798.3 billion yuan, USD 1 = RMB 6.05 yuan as of 1 January 2014) of the national total GDP in 2010. During the first decade of the 21st century, urban built area, average household annual income, and average household living area increased substantially by 2.6, 1.9, and 0.7 times above their 2000 levels, respectively. Car-holding per 100 households increased by 17.1 times, rising from 0.98 to 17.8 cars over the same time. These tremendous changes could have a significant impact on the pattern and behavior of urban households’ energy consumption, aggravating household carbon emissions.

The YRD used 17.5% (or 376.9 mega-tons (Mt) of standard coal) of total energy consumption in China and subsequently produced 15.5% (or 1,228.4 Mt) of national carbon emissions in 2010 [30]. According to the National New-type Urbanization Plan (2014–2020), the country’s first official plan on urbanization, the three largest urban clusters that involve the YRD, Beijing-Tianjin-Hebei and Pearl River Delta in the east coast of China will continue to gain momentum in the process of urbanization and will play pivotal roles in the new era of urbanization, while the development of numerous Third- and Fourth-scaled cities within these clusters will be accelerated [35]. This implies that sustained industrialization and urbanization will continue to foster economic and population growth, undoubtedly leading to a trajectory of growing demand for, and consumption of, energy and other natural resources, goods and services, and consequently increasing carbon emissions in the next two decades if policy initiatives do not target the right populations and/or are poorly implemented.

### Data collection

**Fig 2:**
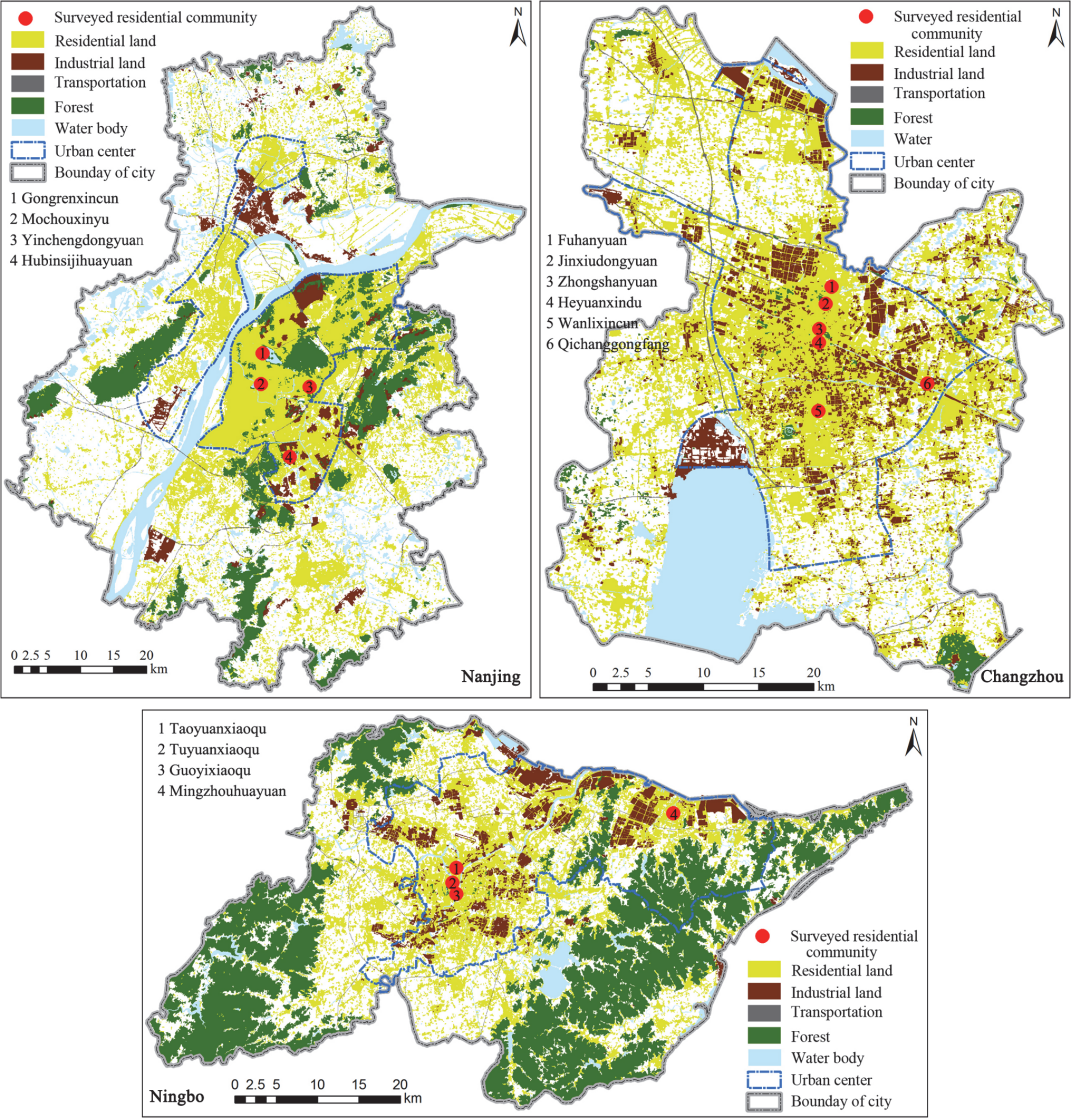
Location and urban land-use types in the residential communities surveyed in the Yangtze River Delta. This is the Fig. 2 legend. Created with the ArcGIS 10.0 software.

The study conducted surveys in the three cities under study to collect specially tailored data related to the primary questions. A *stratified random sampling* method was used to select the residential communities in each surveyed city. Selection criteria considered different contexts of urban communities and households: urban land use, distribution and density of industrial sectors and population, geographical location, household income and the year housing was built (Table 1). As a result, four residential communities in Nanjing and Ningbo respectively, and six communities in Changzhou were selected as representative (Fig. 2). Due to the fact that the numbers of residential communities and residents in the central business district (CBD) of Nanjing are very small, residential communities there were not included in the survey.

**Table 1:** Selection criteria for residential communities surveyed.

**City**	**Residential community**	**Location**	**Housing built year**	**Average annual household income (‘000 yuan)**	**N**
**Nanjing**	1 Gongrenxincun	Inner urban	1950s-1980s	50–100	95
	2 Mouchouxinyu	Inner urban	1985–1990	<50	84
	3Yinchengdongyuan	Inner urban	2003–2007	>100	65
	4 Hubinsijihuayuan	Outer urban	2000–2002	50–100	79
**Ningbo**	1 Taoyuanxiaoqu	Inner urban	1998–2004	>50	141
	2 Tuyuanxiaoqu	CBD	1988	<50	32
	3 Guoyixiaoqu	CBD	1990	50–100	69
	4 Mingzhouhuayuan	Outer urban	2003–2006	>100	60
**Changzhou**	1 Fuhanyuan	Inner urban	2005–2007	>100	88
	2 Jinxiudongyuan	Inner urban	2000–2003	50–100	94
	3 Zhongshanyuan	CBD	1993	50–100	30
	4 Heyuanxindu	CBD	2005	>100	38
	5 Wanlixincun	Outer urban	1995–2000	<50	92
	6 Qichanggongfang	Outer urban	1985–1990	<50	96

A *systematic random sampling* method was used to select urban households to be surveyed. Approximately 350 households in each city were included in the sample (Table 1). The list of the resident households in each residential community, provided by local residents committees, was used as a framework of the population to be sampled. A structured questionnaire survey was conducted through face-to-face interviews in each city in August-October 2011. The content and questionnaire method of the survey were reviewed and approved by each residential community committee covered in our survey in each city (see Fig. 2 and Table 1 for detailed names and locations). A notification about the survey (including a brief introduction of the questionnaire content, purpose and estimated time to complete the questionnaire) was posted on the entrance of each residential building in each surveyed community one week ahead of the survey schedule. Only verbal consent of the participants was obtained because they were very cautious of signing any form of document and obtaining written consent was most likely to cause a high non-response rate. The interviewers read the ethics information to potential respondents and ticked on the consent form if they gave verbal consent. Similarly, we obtained verbal consents from all the residential committees and the ticked (not signed) consent forms were well stored by our research institute. Our overall research has been approved by the Human Research Ethics Committee (HREC) of the Nanjing Institute of Geography and Limnology, Chinese Academy of Sciences (CAS). The HREC of the Institute had approved this consent procedure before we conducted our surveys in the case study areas.

The survey questionnaire comprised four major domains: household demographic and economic characteristics, energy consumption behavior, domestic wastes, and measures adopted (or perceived) to reduce household carbon emissions. The household head answered household level questions involving household size, living area, income, awareness of energy-saving policy and perceived measures for energy saving. The household head, on behalf of other household members, also responded to questions about their basic demographic and economic characteristics including educational attainment, occupation, daily commuting (e.g., commuting time, distance to workplace and transportation means) and long-distance travel (e.g., distance to destination, frequency of travel, and transportation means). If the head was unavailable at the time of the survey, his or her spouse, or an informant in the household who was most knowledgeable about the household situations, answered the survey questions. Information collected about daily commuting for each household member was summed up to annual consumption. Information on average daily garbage generated by a household was collected over a timespan from August to October in 2011, a season shifting from summer to autumn that well represents the normal amount and composition of daily garbage produced in a year. Monthly information on actual energy usages consumed by respondent households was sourced from the corresponding companies providing electricity, water, natural gas, and LPG in each city.

### Survey data

The total sample includes 1,061 households. The survey sample provides a good representation of the target populations. Compared to the 2010 census data, the surveyed households across the three cities are well representative in terms of household employment ratio and per capita living area (Table 2). There is a slight over-representation of urban households with a large number of family members. Nonetheless, because the sample questionnaire was unlikely to collect exact information on household income due to its sensitivity and confidentiality, the questionnaire employed discrete choices of household income bracket (0–50,000 yuan, 50,001–100,000 yuan, 100,001–200,000 yuan, 200,001–300,000 yuan, >300,000 yuan). To make descriptive and regression analyses more meaningful, the ‘income’ variable was treated as a semi-continuous variable by employing the mid-point value of each income bracket (25,000 yuan, 75,000 yuan, 150,000 yuan, 250,000 yuan, 300,000 yuan) as the approximation of household income. The resultant lack of complete accuracy of survey data on income and the mid-point value method used caused wide discrepancies between per capita household incomes in the surveys and their corresponding figures in census. Similarly, average levels of household members’ education in the surveys differ significantly from the average educational attainments of the whole urban population in the 2010 census. The group of households with high educational attainment is over-represented, while the groups holding ‘primary school or below’ and ‘intermediate school certificates’ are under-represented. Additionally, the average age of household members in the survey is older than the mean of the entire population in the census of each city. Thus the analytical methods used may cause some biases. Despite these, the tailored survey information presents a unique insight into the influences of urban households on carbon emission.

**Table 2:** Selected household attributes, by city.

**Mean of household characteristics**	**Nanjing**		**Ningbo**		**Changzhou**	
	**Census 2010**	**Survey**	**Census 2010**	**Survey**	**Census 2010**	**Survey**
**age (years)**	36.6	42.2	36.4	43.7	37.2	41.3
**household size (persons)**	2.7	3.3	2.4	3.0	2.7	3.3
**living area per capita (m** ^**2**^ **)**	29.4	31.9	28.3	30.7	36.5	36.5
**employment ratio** ^**a**^ **(%)**	47.3	48.3	52.3	48.8	52.9	56.6
**annual household income** ^**a**^ **(1,000 yuan)**	76.4	128.7	72.4	118.3	70.9	130.8
**education level (% of total population):**						
**primary schooling or below**	16.2	4.5	29.1	11.0	23.7	6.3
**intermediate school certificate**	50.8	30.2	54.2	43.9	62.1	43.1
**college Diploma or university degree**	33.0	65.3	16.8	45.1	14.2	50.6

Source: 2010 China Census; Statistical Yearbook 2011 of Nanjing; Statistical Yearbook 2011 of Ningbo; Statistical Yearbook 2011 of Changzhou; authors’ survey in 2011.

^a^Employment ratio (%) and annual household income (‘000 yuan) were calculated based on data sourced from statistical yearbooks 2011 for Nanjing, Ningbo, and Changzhou.

### Estimating carbon emissions

Carbon emissions can be computed by multiplying each household’s specific type of energy consumed and its corresponding carbon emission coefficient [10, 33]. This study used a set of carbon emission coefficients suggested by authoritative agencies to estimate urban household carbon emissions in the study area (Table 3). Guided by the 2006 IPCC Guidelines, the method used to estimate urban household carbon emission is expressed in Equation (1).

$$C=\sum_{i=1}^{4}F_{i}*E_{i}+W*E_{w}*\text{365}+\sum_{j=1}^{6}DT_{j}*E_{j}\text{*256}+\sum_{\text{k}=\text{1}}^{\text{4}}LT_{k}*E_{k}*n$$ (1)

Where *C* is the annual total amount of household carbon emissions (kg CO_2_); *i* denotes the energy categories; *F*_*i*_ is the total usage of the *i* ^*th*^ energy (including electricity, water, natural gas and LPG); *E*_*i*_ is the carbon emission coefficient for the *i* ^*th*^ energy; *W* is the average amount of domestic wastes produced per day; *E*_*w*_ is the coefficient of carbon emission for disposing domestic wastes, which is set to the average CO_2_ emission over the 2005–2010 period in Suzhou city of the YRD. The average was calculated based on a survey about the garbage weights, disposal means and carbon emissions (including landfill and incineration) [40]; *j* denotes the transport means for daily commuting; *DT*_*j*_ is the travel distance of specific transport means for daily commuting; *E*_*j*_ is the carbon emission coefficient for the *j* ^*th*^ daily commuting transport means; *256* is the average working days in a year excluding the paid annual leave (days); *k* denotes the long-distance transport means; *LT*_*k*_ is the travel distance of the *k* ^*th*^ long-distance transport means; *E*_*k*_ is the carbon emission coefficient for the *k* ^*th*^ long-distance transport means; *n* is the number of long-distance trips in a year.

**Table 3:** Coefficients for estimating urban household carbon emissions.

Domain	Coefficient	Unit	Explanation	Source
**Energy consumption:**				
**Electricity**	0.96	kg CO_2_/kWh	A kWh of electricity yields 0.96kg CO_2_.	Ministry of Science and Technology (MST), China [36]
**Water**	0.3	kg CO_2_/tons	Including energy consumed for operating water processing plants and sewage treatment plants.	MST [36]
**Natural gas**	2.19	kg CO_2_/m^3^		Wang et al. [37]
**LPG**	2.84	kg CO_2_/kg		Wang et al. [37]
**Transport means:**				
**Foot/bicycle**	0	kg CO_2_/km		
**Electrical bicycle**	0.022	kg CO_2_/km	20 mA 48V electromobile uses 1.13 kWh electricity for each charge, which can drive 50km.	MST [36]
**Bus**	0.0555	kg CO_2_/km		Zhang Q et al. [38]
**Motorcycle**	0.075	kg CO_2_/km	A liter of petrol fuels drives 30km.	MST [36]
**Subway**	0.945	kg CO_2_/time	Average electricity consumption for single subway is 1.19 kWh.	Xie et al. [39]
**Car**	2.34	kg CO_2_/L		MST [36]
**Long-distance bus**	0.019	kg CO_2_/km	Fuel consumption is estimated at the rate of 30 liters for 100 km on the basis of a 45-seat long-distance coach.	MST [36]
**Train**	0.062	kg CO_2_/km		GHG Protocol [20]
**Aircraft**	0.18	kg CO_2_/km	Energy efficiency differences between long, medium and short routes are not differentiated.	Conservation International [20]
**Garbage disposal:**				
**Domestic wastes**	2	kg CO_2_/kg	Including waste incineration and landfill but excluding recycling.	Zhang T et al. [40]

### Independent variables

A range of factors influencing household carbon emissions are grouped into four categories: demographic factors, economic factors, behavioral/cognitive factors, and spatial factors. *Demographic factors* regarding household head’s age and educational attainment, household’s gender composition, family size, and dependency ratio are used to measure the key demographic characteristics of a household as these factors combine together to shape the household’s experience and ability to reduce carbon emission. In addition to total income, a particular interest of this study is to examine how the *economic factors* of a household—employment ratio, different occupations of household heads, car-holding, and total living area of the household—could influence the households’ carbon emissions. *Behavioral/cognitive factors* in this study mainly involve the awareness of household energy saving and carbon emission reduction, useful measures perceived by households to reduce CO_2_, and temperature set for air conditioning in summer. Specifically, the questionnaire asked respondents if they were aware of the terminology of ‘household energy saving’ and ‘carbon emission reduction’ (yes/no). The questionnaire also asked them ‘to what temperature do you set your home air conditioning in summer?’ Three options were for their choice: below 26°C, 26°C, and over 26°C. Another question put to them was ‘what measures do you consider being useful for your family to save expenses on energy and then reduce carbon emission?’ Specific measures include reducing usage of electricity, reducing usages of water and gas, reducing the frequency of driving a family car, reducing purchases of unnecessary goods, and reducing food wastes. For each measure perceived by the respondent, the factor ‘measure perceived’ is indexed as 1, yielding a total score of this factor ranging from 0 to 5. The list of independent variables entered into the regression and their definitions are presented in Table 4.

**Table 4:** Definitions of independent variables.

**Variables**	**Definitions**
**Demographic factors**	
**age**	age of household head
**age squared**	Age squared
**hh_size**	the number of household members
**male_ratio**	the ratio of males relative to the total number of the household members: [0,1]
**dependency ratio**	the ratio of those not at the working age (aged 15 years or younger, or 60 years or over) against the total number of household members: [0,1]
**education**	the highest educational attainment of household head: 1 = primary schooling or below; 2 = intermediate school certificates; 3 = college Diploma or university degree
**Economic factors**	
**income**	household’s total annual income: [25, 75, 150, 250, 300] ('000yuan)
**employment_ratio**	the ratio of persons employed against the total number of household members: [0,1]
**occupation**	the occupation of household head: 1 = if the household head has a high-end occupation such as government officials, public servants, professionals or associate professionals in financial/legal/medical institutions; 2 = if the household head has an occupation as a worker in manufacturing industry; 3 = if the household head is self-employed; 4 = if the household head has an occupation as a tradesperson or similar, such as advanced/intermediate clerical, sales and service workers; 5 = if the household head is retired, or is a student, farmer or other
**car**	household’s car-holdings: 0 = if the family has no car; 1 = if the family has at least 1car.
**living_area**	total living area of the household (m^2^)
**Behavioral (cognitive) factors**	
**energy_saving**	the temperature of air conditioning set in summer: 1 = below 26°C; 2 = 26°C s; 3 = over 26°C
**energy_awareness**	1 = if the respondent is aware of the terminology of ‘household energy saving’ and ‘carbon emission reduction’; 0 = otherwise
**measure_perceived**	the number of useful measures perceived by the household to reduce CO_2_: [0,5]
**Spatial factors**	
**city**	1 = if the household is in Nanjing; 2 = Ningbo; 3 = Changzhou
**distance_cbd**	the distance from residence of a household to the CBD (km)

### ANOVA test and regression analysis

In order to explore as widely as possible the relationships between household carbon emission and various household characteristics, correlation analysis was applied for continuous independent variables (e.g., age, male ratio, household size, dependency ratio, employment ratio, living area, and number of perceived useful means for energy saving). ANOVA test was used to examine whether average household carbon emissions vary by household specific characteristic that can be categorized into multiple groups (e.g., education, occupation, income, car-holding, energy-saving behavior, awareness of energy saving, and residency location). OLS regression was employed to analyze the significance and weights that multiple factors may contribute to household carbon emissions. Both factors of ‘energy-saving awareness’ and ‘perceived energy-saving means’ are excluded in the regression though they have positive association with CO_2_ emissions. Greater energy-saving awareness and more energy-saving measurements are not significantly related to less energy use of households. So there is no clear causal relationship between these two variables and CO_2_ emission.

The numerical amount of CO_2_ emission is transformed using the natural logarithm function, so that the data appears to more closely meet the assumptions of a statistical inference procedure of OLS models. The OLS procedures involve backward step by step removing of the least significant independent variables until all the remaining independent variables are at least significant at the 10 percent significance level. The advantage of this procedure is that the final model would have fewer irrelevant independent variables and therefore minimize the standard errors of the estimates of the remaining independent variables. The disadvantage is that the final model may suffer from omitted variable bias if any dropped variable is fundamental to explaining the dependent. Our model test proves that no significant variable was omitted.

## Results

### Magnitude of household carbon emissions

The average of annual urban household carbon emissions in the three cities was estimated to be 5.96 tonnes CO_2_ (Table 5). Household carbon emissions originated from four major domains—energy consumption, daily commuting, garbage disposal and long-distance travel—against the total household emissions in the three cities account for 51.2%, 21.3%, 16.0% and 11.5%, respectively. Electricity consumption is the largest single contributor (at 86.8%) of the total carbon emissions from all types of domestic energy consumption. Average carbon emission derived from household daily commuting almost doubles that generated from long-distance travel. The mode of transport has an important impact on carbon emissions. Specifically, average carbon emission derived from household daily commuting by family car (2.66 tonnes CO_2_ per household) equals 8.4 times that generated by bus and 5.9 times that generated by subway. Average carbon emissions from household long-distance travel by airplane (2.06 tonnes CO_2_) are 28.8 times that by coach, 6.9 times that by family car, and 6.5 times that by train. These figures suggest that energy consumption and people’s choices among transport modes are dominant factors influencing carbon emissions at the household level. This result is consistent with other studies [41, 42].

**Table 5:** Average urban household carbon emissions (tonnes) in Nanjing, Ningbo and Changzhou in 2010.

**Source of carbon emissions**	**Nanjing**			**Ningbo**			**Changzhou**			**Total**		
	**Mean**	**Std. Dev.**	%	**Mean**	**Std. Dev.**	%	**Mean**	**Std. Dev.**	%	**Mean**	**Std. Dev.**	%
**Energy consumption:**												
**Electricity**	2.94	2.21	46.2	2.31	1.17	43.7	2.68	1.96	43.7	2.65	1.87	44.4
**Water**	0.04	0.02	0.6	0.03	0.02	0.5	0.03	0.02	0.5	0.03	0.02	0.6
**Gas**	0.50	0.71	7.8	0.25	0.16	4.8	0.36	0.23	5.8	0.37	0.44	6.2
**Sub-total**	3.47	2.49	54.6	2.59	1.21	49.0	3.07	2.09	50.0	3.05	2.05	51.2
**Transportation**												
**Daily commuting**	1.37	1.76	21.58	1.12	1.64	21.3	1.30	1.83	21.2	1.27	1.76	21.3
**Long-distance travel**	0.54	1.47	8.6	0.56	1.14	10.6	0.87	2.21	14.2	0.69	1.75	11.5
**Sub-total**	1.91	2.54	30.1	1.68	2.17	31.9	2.17	3.28	35.4	1.95	2.79	32.8
**Garbage disposal**	0.97	0.48	15.3	1.01	0.30	19.1	0.90	0.36	14.6	0.95	0.39	16.0
**Per household**	6.36	4.19	100.0	5.28	2.71	100.0	6.14	4.40	100.0	5.96	3.94	100.0
**Per capita**	2.03	1.31		1.89	1.02		1.92	1.23		1.94	1.20	

Source: authors’ estimation based on survey data and the simulation model expressed in Equation (1).

Energy policy has a great effect on household energy consumption and resultant carbon emissions. Household electricity usage (average 2,403.7 kWh) in Ningbo was less than the average levels in Nanjing (3,057.7 kWh per household) and Changzhou (2,791.5 kWh per household). The disparity is mainly caused by different policies on usage charges in these cities. As one of China’s pilot cities to explore pathways for solving power shortages, Ningbo has applied different rates for electricity use to households since 2004. In comparison, Nanjing and Changzhou did not commence a similar policy until July 2012, when China began to implement a household ‘incline block tariff’ designed as the initial step towards a carbon tax. Under the policy practiced in Ningbo, electricity used beyond a certain limit has been charged at higher rates. In 2010, electricity use in Ningbo was charged at the rate of 0.588 yuan/kWh for usage between 2761–4800 kWh and at a much higher rate of 0.838 yuan/kWh for the band of usage over 4800 kWh. These rates were higher than the pricing implemented in Changzhou (by 55.8%) and in Nanjing (by 9.3%). High rates for electricity use in Ningbo led to less household carbon emissions from electricity than in Nanjing and Changzhou in 2010, by 0.63 and 0.37 tonnes CO_2_, respectively.

### Impact factors

The results of correlations between household carbon emissions and key continuous variables are presented in Table 6. The factors of family living area, age, age squared, household size, dependency ratio, employment ratio, and number of perceived means useful for carbon reduction have statistically significant and relatively strong correlations with CO_2_ emissions. The highest absolute Pearson coefficients for correlations between demographic and other independent variables are less than 0.67 at the 5 percent significance level, indicating little risk of multicollinearity problems should they be included in the regression model.

**Table 6:** Pearson correlation coefficients for household CO_2_ emissions and selected household factors.

	**age**	**age squared**	**hh_size**	**male_ratio**	**dependency**	**Employment_ratio**	**living_area**	**Perceived_measures**	**distance_cbd**
**household CO**_**2**_ **emissions**	-0.257***	-0.268***	0.287***	-0.041	-0.139***	0.164***	0.494***	0.084***	0.054*
**age**		0.988***	-0.266***	0.074**	0.574***	-0.53***	-0.256***	-0.040	-0.106***
**age squared**			-0.275***	0.066**	0.615***	-0.559***	-0.262***	-0.044	-0.113***
**hh_size**				-0.155***	-0.156***	0.008	0.213***	-0.026	0.010
**male_ratio**					0.121***	0.031	-0.008	0.062**	0.004
**dependency**						-0.668***	-0.091***	0.038	-0.077**
**employment_ratio**							0.149***	0.051	0.098***
**living_area**								0.07**	0.222***
**perceived_measures**									-0.043

*p<.10;

**p<.05;

***p<.01.

The *behavioral factor* related to ‘perceived measures’ is found to be positively significant with household carbon emission (Table 6). The factor of ‘awareness’ is also associated with household CO_2_ emission but at a lesser significant level (Table 7). It is paradoxical with more perceived carbon mitigation measures and higher awareness resulted in higher emissions. This result contrasts sharply with the expectation of the Chinese government which anticipates that governmental policies and programs regarding carbon mitigation and energy saving could play an effective part in reducing CO_2_ emissions. Clearly, there is a marked mismatch between perception of carbon reduction measures and actual household energy consumption behavior. This also implies that the real awareness of energy-saving and carbon reduction in the YRD is still at the lower level. Therefore, both ‘energy-saving awareness’ and ‘perceived means’ are excluded in the regression as independent variables. How to enhance people’s low-carbon awareness and subsequently adjust their behavior in actual energy consumption exhibits a tremendous challenge, especially in the YRD which is moving toward a rapid transition for urban households to change from middle to high income conditions. This must be a significant area of policy concern.

**Table 7:** Statistics and *P*-values of ANOVA tests of CO_2_ emissions of urban households categorized by selected household characteristics.

**Categorical factors**	**Mean**	**Std. Dev.**	**Freq.**
**Car-holding**			
**0**	4.22	2.14	632
**1**	8.40	4.07	428
**Total**	5.91	3.69	1060
***F = 307*.*79*, *P = 0*.*000***			
**Annul income**			
**25 ('000yuan)**	3.62	1.59	216
**75 ('000yuan)**	5.22	2.86	318
**150 ('000yuan)**	6.30	2.93	283
**250 ('000yuan)**	8.10	4.39	123
**300 ('000yuan)**	10.04	5.39	95
**Total**	5.97	3.70	1035
***F = 85*.*32*, *P = 0*.*000***			
**Energy saving**			
**setting air-conditioning temperature lower than 26°C**	7.38	4.37	148
**26°C**	6.13	3.65	204
**higher than 26°C**	5.55	3.30	690
**Total**	5.92	3.59	1042
***F = 16*.*73*, *P = 0*.*000***			
**Awareness of energy-saving**			
**without awareness**	5.58	3.66	349
**having awareness**	6.08	3.65	688
**Total**	5.91	3.66	1037
***F = 4*.*35*, *P = 0*.*037***			
**Education**			
**primary school or below**	4.78	2.70	202
**junior to senior high school**	5.82	4.00	357
**advanced Diploma or university degrees**	7.12	3.88	345
**Total**	6.08	3.81	904
***F = 27*.*16*, *P = 0*.*000***			
**Occupation**			
**high-end occupation**	8.17	4.85	197
**manufacturing workers**	6.54	3.78	187
**self-employed**	6.05	3.55	116
**tradespersons, clerical/sales/service workers**	5.32	2.77	215
**retired, student, farmer or others**	4.56	2.59	323
**Total**	5.93	3.71	1038
***F = 35*.*98*, *P = 0*.*000***			
**City**			
**Nanjing**	6.36	4.19	322
**Ningbo**	5.31	2.72	302
**Changzhou**	6.00	3.83	437
**Total**	5.91	3.69	1061
***F = 6*.*57*, *P = 0*.*001***			

The small *p*-values suggest that the mean of household CO_2_ emissions among different households categorized in terms of car-holding and household income are both significantly different (Table 7). This result is consistent with the findings of other researchers [17–20]. Table 7 also suggests that the lower the temperature is set for air conditioning in summer, the greater the amount of CO_2_ that the household emits. There is a significant difference among the average CO_2_ emissions from three types of households categorized by household head’s highest level of educational attainment. The higher the education level, the more carbon emission of the household. Similarly, if the household head works in a high-end occupation, the family seems to emit more carbon than those households in which household heads work in intermediate and low-end occupations. Finally, households residing in Nanjing emit more carbon than their counterparts living in Changzhou and Ningbo.

Table 8 shows the results of the initial and final models estimated with robust standard errors to minimize heteroscedasticity. The final model well predicts CO_2_ emissions in the study area, as demonstrated by the high value of R-squared (0.534), the linearity of the model and the behavior of the residuals. A set of demographic factors significantly, and positively, influence household carbon emissions. The magnitude of carbon emissions will increase by 10.7% for a one unit increase in the dependency ratio of a household. Carbon emission is likely to increase by 7.0% for a one person increase in the household members, assuming that other factors remain unchanged. Also carbon emission will grow slightly with people getting older, at a rate of 1.8% growth for one year increase in age until one reaches a certain age and then shifting to decline in carbon emission by age. Surprisingly, ‘educational attainment’ of household head has statistically insignificant associations with carbon emissions if other factors remain to their mean. This finding seems to be contrary to some other studies [11, 12]. The result suggests that the highest educational level could be an endogenous factor as it could directly and significantly influence other household characteristics such as income, occupation, employment ratio, car-holding and living area.

**Table 8:** OLS regression results: factors influencing urban household carbon emissions.

**Variables**	**Initial model (1)**	**Final model (2)**
	**Coef.**	**Coef.**
**Demographic factors**		
**age**	0.019***	0.018**
**age squared**	-0.000***	-0.000***
**male_ratio**	0.011	
**hh_size**	0.076***	0.070***
**dependency ratio**	0.127**	0.107**
**education (baseline: primary school or below)**		
**junior to senior high school**	-0.005	
**advanced Diploma or university degrees**	0.035	
**Economic factors**		
**employment_ratio**	0.059	
**occupation (baseline: retired, student, farmer or others)**		
**high-end occupation**	0.06	
**manufacturing workers**	-0.016	
**self-employed**	-0.037	
**tradespersons, clerical/ sales/service/workers**	-0.024	
**income (baseline: yearly income 0–50k yuan)**		
**50–100k yuan**	0.105***	0.115***
**100–200k yuan**	0.186***	0.204***
**200–300k yuan**	0.259***	0.287***
**>300k yuan**	0.318***	0.352***
**car**	0.462***	0.474***
**living_area**	0.002***	0.002***
**Behavioral (cognitive) factors**		
**energy_saving (baseline: < 26°C)**		
**26°C**	-0.151***	-0.141***
**>26°C**	-0.146***	-0.145***
**Spatial factors**		
**city (baseline: Changzhou)**		
**Nanjing**	0.147***	0.150***
**Ningbo**	0.054*	0.056*
**distance_cbd**	-0.005***	-0.006***
**_cons**	7.338***	7.433***
**Observations**	866	866
**R-squared**	0.538	0.534

*p<.10;

**p<.05;

***p<.01.

*Economic factors* are related to household carbon emissions, statistically significant and quantitatively substantial (Table 8). Household ‘car-holding’ contributes overwhelmingly to CO_2_ emission. Compared to those having no car, the households possessing 1 or more cars will emit more carbon by 47.4% if other factors remain unchanged. The ‘income’ level influences CO_2_ emissions enormously. The higher annual income the household has, the greater carbon it will generate. For example, carbon emissions would raise by 11.5% for a household with annual income being within the 50,000–100,000 yuan bracket, compared to the baseline group of households with annual income less than 50,000 yuan. The average percentage will further increase by about 28.7% for households with incomes ranging between 200,000–300,000 yuan, and further by 35.2% for high income (over 300,000 yuan) households. ‘Living area’ contributes to carbon emission significantly, growing by 0.2% of CO_2_ for every 1 m^2^ of increase in the living area. There is no statistical evidence showing that the occupational level or employment proportion of the household contributes to household carbon emissions.

The *behavioral factor* related to ‘energy saving’ is found to be statistically significant at the one percent significance level and greatly related to urban household carbon emissions (Table 8). If ‘energy saving’ changes from the baseline level (i.e., setting the temperature of air conditioners below 26°C in summer) to a higher level at which temperature of air conditioners is set to 26°C or over, the household carbon emissions will decline by approximately 14.5%.

‘Geographical location’ is significantly correlated with carbon emissions for urban households (Table 8). Households living in Nanjing will produce more carbon (by 15.0%) than their counterparts in Changzhou, while the rate of increase will be on a smaller scale (5.6%) in Ningbo and on a weaker significance level assuming other factors remain unchanged. This is not surprising as the effect of the ‘distance to CBD’ is greatly mediated by daily commuting from home to workplace and transportation means. Households residing in Heyuanxindu and Zhongshanyuan communities in Changzhou, located near the CBD, provide an example in point. Interestingly, the minimum household carbon emissions from daily commuting (0.39 tonnes) appeared in Qichanggongfang community of Changzhou city (numbered 6 in Fig. 1). This community is a satellite town being 13km away from the CBD. The community functions as both a residential and industrial district, where many people take local jobs in large state-owned heavy industries. Short distance of commuting to workplace enables workers to take an electric bicycle (44.8%) and foot/bike (31.2%) as the dominant means of commuting, while driving cars or taking buses accounts for a small percentage (10.4%). Note that daily commuting contributes the second biggest proportion (21.3%) to household carbon emissions in the three cities. This finding suggests that this type of urban zoning can facilitate reduction of household carbon emissions.

### Policy implications

With an anticipated urbanization rate of 75% (compared to the overall national urbanization rate of about 60%) and rapid economic development in the YRD by 2020 [43], continuing growth in urban population due to rural-to-urban migration and natural growth, aging of the population, increasing car-holding, household income and living area are expected to push household carbon emissions to higher levels in the next decade. Assuming that on average each urban household will own one car, have total income with 250,000 yuan (which almost double their corresponding levels in 2010), and have per capita living area of 35m^2^ by 2020, we estimated that household carbon emission will increase by 1.4 tonnes CO_2_ (or by 26.1%), based on the final model. Further, we estimated that 14.5% of total increase in household carbon emission induced by the growth of household income, family car-holding, and living area could be offset by lifting household energy-saving means to the most efficient mode (i.e. setting home air conditioning at temperature over 26°C in summer). This is especially the case in First- and Second-tier cities such as Nanjing and Ningbo examined in this study. It is of great significance to take practical countermeasures to control or even reduce household carbon emissions to achieve the regional target of carbon reduction by 2020.

Household car-holding is found to be a very important factor that contributes to carbon emissions at the household level. Thus regulating family car-holdings should be taken as the highest policy priority for policy making and urban planning. To achieve an equilibrium between automotive industry development, increasing demand for family cars, and national/regional targets for energy-saving and carbon mitigation, economic levers in combination with mandatory measures need to be in place and work together. Economic levers include: (1) car license-plate auctions, as practiced in Shanghai; (2) different purchase taxes and environmental pollution taxes levied on different types of cars in terms of fuel consumption or the number of engine cylinders. Mandatory measures, such as restricting car numbers by daily rotation of on-road use between even- and odd-digit license numbers and releasing license-plates through lottery, which have been implemented in megacities such as Beijing and Guangzhou, can be applied to First- and Second-tier cities in the delta region. These measures have been demonstrated to be effective ways to control the rapid growth of both numbers and composition of family cars in Shanghai [44] and in other cities [7]. Setting a cap for annual car additions or increasing the costs for driving cars in order to slow down growing demand for family cars has encountered some critical controversy in the current phase of fast economic, demographic and social transitions in China. To lower household emissions, local governments need to optimize urban planning and public transport networks, develop highways and subways, and direct urban citizens to take public transport.

The average household size in the YRD declined from 2.94 persons in 2000 to 2.73 person in 2010 due to the sustained implementation of national family planning policy since 1979. Nevertheless, the total population grew rapidly over the same time period, increasing by 27.42 million (or 34.2%). According to population censuses in China, the dependency ratio in the urban areas across 16 major cities of the YRD experienced a downward trend over the decade to 2010, declining from 26.4% in 2000 to 23.2% in 2010 due mainly to massive inflows of inter-provincial migrants to the deltaic region. This is compared with an upward trend of population aging in the region, with a rate of population aging (aged 60 years or older) changing from 12.6% to 13.1% over the same period. To combat China’s fast population aging and to ensure coordinated economic, social and population development, the Chinese government has reformed its decades-long one-child family planning policy, allowing a couple to have two children if one of the couple was born as a single child [45]. This significant shift will affect 15–20 million young people born after 1979. The implementation of this new policy can be expected to slightly stimulate natural population growth and household size in the future. Therefore, the increasing carbon emission associated with possibly growing household size, rising dependency of the elderly and young groups in the working-age population, and growing population would increase household carbon emissions in the YRD and China in the next decade if other factors remain constant.

With the implementation of the National New-type Urbanization Plan (2014–2020), growth in the middle income class in the YRD will be undoubtedly accelerated. As a result, carbon emissions will undoubtedly increase when household income goes up. Advocating low-carbon living styles and improving their low-carbon consumption behavior are important steps for people to shift from planned or intended behavior (i.e., recognizing the problem of carbon emission and the necessity to solve it) to actual behavior (i.e., adopting energy-saving measures). The study shows robust and strong evidence that setting air conditioning temperature to a higher degree than 26°C in summer (and conversely to a lower temperature in winter) can significantly reduce carbon emissions. However, the trend for a growing demand for family cars, increasing income and living areas, and a rising number of affluent urban households over the next two decades cannot be reversed in the phase of China’s fast development. Thus, improving energy-saving measures and transforming luxurious lifestyles into low-carbon living styles are suggested as imperative countermeasures for carbon reduction at the household level. Transition in urban household living styles suggested in this paper was also echoed in other studies [9].

## Discussion

The estimated average of urban household carbon emissions in Nanjing (6.36tonnes) in 2010 in this paper was greater (by 72%) than that estimated by Yang et al.[20]. The estimation of the latter study was based on survey data from only three low-income (less than 55,669 yuan) communities in Nanjing. Their study captured some characteristics of a narrow segment of urban population. Their estimated average of carbon emissions was similar to our estimate (3.62 tonnes) for the low income group of households (less than 50,000 yuan) in Nanjing. Our study reflected a full income spectrum of diverse households living in different urban settings across three major cities. This enabled us to better analyze the effects of important household characteristics—family car-holding, income level, energy saving behavior, household size and age structure (measured as dependency ratio), and living area—on household energy consumption and carbon emissions. Strikingly, per capita carbon emissions generated from urban residents’ energy use (including electricity, water and gas) in this study was far greater (by 91%) than the figure estimated by Wang [8]. Wang’s estimation was computed by averaging out household carbon emissions in Shanghai municipality and provinces of Jiangsu and Zhejiang, using statistical data sourced from the Chinese Energy Statistics Yearbook. Due to the inherent limitations in the statistical data at the provincial level carbon emissions could be underestimated. Moreover, the jurisdictional boundary of the three municipality/provinces in the YRD (Shanghai, Jiangsu and Zhejiang) is broader than the YRD used in this paper (defined as an economic and geographic region). The former boundary encompasses some less developed cities in Jiangsu and Zhejiang, leading to an underestimation of carbon emissions.

Table 9 summarizes the differences of primary factors to household carbon emissions compared with other similar studies at the micro scale. Key factors identified in our study are similar to many other studies, including household income, housing area, household size and age structure. Some factors are identified to be specific primarily due to different scope and context of research [13, 18, 19]. Generally, dominant contributing factors in this study are consistent with other studies. An exception is that relationship between education and household carbon emission in this study was not significant, which contradicted with some other research [11]. Generally, examining household carbon emissions using survey data at the micro (household) level can precisely capture the interrelationships between household carbon emissions and various demographic, economic, behavioral/cognitive, and spatial factors.

**Table 9:** Differences of contributing factors between this study and other related studies at the micro scale.

**Studies**	**Study Area**	**Contributing factors**	**Data collection**
**This study**	YRD, China	Family car-holing, income, energy-saving awareness, household size, housing area, dependency ratio, distance-CBD, age structure	Household energy and transport survey of 1,061 households in Nanjing, Ningbo and Changzhou in August-October 2011
**Baiocchi et al. [11]**	United Kingdom	Household income, education, lifestyles, household type, internet usage	2001 census, CACI’s consumer lifestyle databases
**Büchs & Schnepf [12]**	United Kingdom	Household size, household income, education, gender, rural location	Household expenditure data of 24,446 households in 2006–2007
**Dalton et al. [13]**	USA	Population aging, technical change	Consumer Expenditure Survey (CES) of households in the U.S.
**Lin et al. [17]**	Xiamen, China	Housing area, household income, household size, building age, marital status	Household consumption survey of 714 households in 2009
**Weber & Matthews [18]**	USA	Household income, expenditure pattern	Consumer expenditures survey of 25,000 households in the USA in 2004
**Golley & Meng [19]**	China	Household income	China's urban household income and expenditure survey (UHIES) in 2005
**Yang et al. [20]**	Nanjing, China	Household size, transportation means, housing area, household income	Household energy and transport survey of 1000 households from May 2008 to May 2009

The limitations of the study deserve mention. Firstly, this study did not consider indirect energy consumption due to the low accuracy of such information from the questionnaire survey. Carbon emission from indirect energy consumptions (e.g., food, consumer items, housing operations, entertainment and services) is estimated to be 2.5–3.4 times more than that from domestic direct energy use (including electricity, water, natural gas, and LPG) in China [6, 8]. It is of significance that carbon emissions generated by other indirect energy consumptions should be taken into account to have a comprehensive understanding of the impact mechanisms for carbon emissions at the household level in future studies.

Secondly, the coefficients used in the estimation model impact on the estimated magnitude of CO_2_ significantly. If the specific carbon emission coefficients for domestic energy electricity (0.926kg CO_2_/kWh), natural gas (2.184kg CO_2_/m^3^) and LPG (2.841kg CO_2_/kg) for China derived from the International Energy Agency were applied to this study [46], urban household carbon emissions of domestic energy use would decrease by 7.3% in Ningbo, 5.7% in Nanjing and 5.1% in Changzhou. It is important to estimate carbon emissions at a high accuracy to make fair international climate negotiations and climate change adaptation policies on all scales. A standard system of rational carbon emission coefficients tailored for specific areas needs to be established.

Thirdly, our analyses are cross-sectional because of the nature of our primary data. The high income and educational attainments of the households surveyed in this study suggest that they were mainly locals. Also the sample size in each city under study is small, thus it is difficult to distinguish between established local urban households and rural-urban migrant households. Addressing different patterns of carbon emissions between such differing household categories is important as they represent different levels of mitigating capacity. Up to the present, no attempts have been made to systematically model and evaluate how migrant households may influence carbon emissions in cities and how and with what effects their mitigating efforts intersect with their urban citizenship, green urbanism, and widening disparities between rich and poor in rapidly urbanizing cities in the YRD. This is an extremely urgent issue given that urbanization is one of the most profound demographic and social processes facing the YRD and China today.

Fourthly, the characteristics of household carbon emissions and key factors for households living in the Fourth-tier (small) cities in the YRD were not discussed in the paper. Consumption behavior, compositional distribution of carbon emissions and factors influencing carbon emissions of households living in this type of cities need to be examined in future research. This is because urbanization in small cities will gain momentum because of the fast development anticipated in the next decade according to China’s current urbanization policy [35, 47]. Despite these limitations, the study presented here offers an important contribution to the burgeoning literature on the nexus of climate change and carbon mitigation.

## Conclusion

Carbon emission from urban households is an important contributor to overall carbon emissions and an integral part of carbon mitigation on the national, regional and municipal scales. The main contribution in this study is an increased understanding of the quantity and mechanisms for carbon emissions on the household scale in the Yangtze River Delta (YRD) of China by using primary data that address key demographic, economic, behavioral/cognitive and spatial factors. This study estimates that average urban household carbon emissions in the YRD amounted to 5.96 tonnes CO_2_ in 2010. Energy consumption, daily commuting, garbage disposal and long-distance travel are identified to be major sources of household carbon emissions. A set of demographic, economic, behavioral, and spatial factors are key determinants of urban household carbon emissions in the region. The age structure (i.e., dependency ratio), household size, income and family car-holding influence household carbon emissions significantly. Fast demographic transition (including rapid urbanization and population growth, and population aging) in the region, as elsewhere in China, will impose great challenges for carbon emissions reduction at the household level over the next two decades.

Carbon emissions of urban households in the YRD can be expected to maintain an increasing trend with the ongoing process of urbanization and economic development in the next decade. It is of great significance and urgency to take action to control carbon emissions, given the irreplaceable strategic significance of this region to maintain the sustainability of economic development and mitigate carbon emissions in China. Practical countermeasures include: regulating the dramatic growth of family cars by incorporating economic levers with administrative measures; controlling fast population growth; and transiting residents’ low-carbon awareness to household behavior in energy and other spheres of consumption. Only by addressing household carbon emission factors and by incorporating fast changing urban household consumption behavior in rapidly demographic and socio-economic transitions into regional development trajectory will it be possible to achieve effective carbon mitigation in the Yangtze River Delta and in China.

## References

[1] Intergovernmental Panel on Climate Change (IPCC). Climate Change 2014: Synthesis Report. Available:http://www.ipcc.ch/pdf/assessment-report/ar5/syr/SYR_AR5_LONGERREPORT.pdf. Accessed 17 December 2014.

[2] Olivier JGJ, Janssens-Maenhout G, Muntean M, Peters JAHW (2013) Trends in Global CO_2_ emissions: 2013 report PBL Netherlands Enviornmental Assessment Agency, Hague.

[3] The White House (2014). U.S.-China Joint Announcement on Climate Change. Available: http://www.whitehouse.gov/the-press-office/2014/11/11/us-china-joint-announcement-climate-change. Accessed 10 December 2014.

[4] Guan DB, Hubacek K, Weber CL, Peters GP, Reiner D (2008) The drivers of Chinese CO_2_ emissions from 1980 to 2030. Global Environmental Change: Human and Policy Dimensions 18: 626–634.

[5] Feng KS, Hubacek K, Guan DB (2009) Lifestyles, technology and CO_2_ emissions in China: a regional comparative analysis. Ecological Economics 69: 145–154.

[6] Feng ZH, Zou LL, Wei YM (2011) The impact of household consumption on energy use and CO_2_ emissions in China. Energy 36: 656–670.

[7] Qi Y (2012) Annual Review of Low-Carbon Development in China (2011–2012). Beijing: Social Sciences Academic Press (in Chinese).

[8] Wang Z (2012) Study on calculation and impact factors of carbon emissions from residents' consumption. Dr. Dissertation, University of Science and Technology of China (in Chinese).

[9] Wei YM, Liu LC, Fan Y, Wu G (2007) The impact of lifestyle on energy use and CO_2_ emission: an empirical analysis of China’s residents. Energy Policy 35: 247–257.

[10] Dietz T, Gardner GT, Gilligan J, Sternd PC, Vandenberghe MP (2009) Household actions can provide a behavioral wedge to rapidly reduce US carbon emissions. Proceedings of the National Academy of Sciences of the United States of America 106: 18452–18456. doi: 10.1073/pnas.0908738106 19858494 10.1073/pnas.0908738106 PMC2767367

[11] Baiocchi G, Minx J, Hubacek K (2010) The impact of social factors and consumer behavior on carbon dioxide emissions in the United Kingdom. Journal of Industrial Ecology 14: 50–72.

[12] Büchs M, Schnepf SV (2013) Who emits most? Associations between socio-economic factors and UK households’ home energy, transport, indirect and total CO_2_ emissions. Ecological Economics 90: 114–123.

[13] Dalton M, O'Neill B, Prskawetz A, Jiang LW, Pitkin J (2008) Population aging and future carbon emissions in the United States. Energy Economics 30: 642–675.

[14] Chancel L (2014) Are younger generations higher carbon emitters than their elders? Inequalities, generations and CO_2_ emissions in France and in the USA. Ecological Economics 100: 195–207.

[15] Druckman A, Buck I, Hayward B, Jackson T (2012) Time, gender and carbon: a study of the carbon implications of British adults’ use of time. Ecological Economics 84: 153–163.

[16] Meier T, Christen O (2012) Gender as a factor in an environmental assessment of the consumption of animal and plant-based foods in Germany. The International Journal of Life Cycle Assessment 17: 550–564.

[17] Lin T, Yu Y, Bai X, Feng L, Wang J (2013) Greenhouse gas emissions accounting of urban residential consumption: A household survey based approach. PLoS ONE 8: e55642 doi: 10.1371/journal.pone.0055642 23405187 10.1371/journal.pone.0055642 PMC3566040

[18] Weber CL, Matthews HS (2008) Quantifying the global and distributional aspects of American household carbon footprint. Ecological Economics 66: 379–391.

[19] Golley J, Meng X (2012) Income inequality and carbon dioxide emissions: the case of Chinese urban households. Energy Economics 34: 1864–1872.

[20] Yang XM, Ge YS, Zeng HY (2010) The household carbon emission analysis under individual consumer behavior. China’s Population Resources and Environment 20:35–40 (in Chinese).

[21] Davis J, Sonesson U, Baumgartner DU, Nemecek T (2010) Environmental impact of four meals with different protein sources: case studies in Spain and Sweden. Food Research International 43: 1874–1884.

[22] Vieux F, Darmon N, Touazi D, Soler LG (2012) Greenhouse gas emissions of self-selected individual diets in France: changing the diet structure or consuming less? Ecological Economics 75: 91–101.

[23] Kim T, Kim H (2013) Analysis of the effects of intra-urban spatial structures on carbon footprint of residents in Seoul, Korea. Habitat International 38: 192–198.

[24] Ehrlish PR, Holdren JP (1971) Impact of population growth. Science 171: 1212–1217. 5545198 10.1126/science.171.3977.1212

[25] Bin S, Dowlatabadi H (2005) Consumer lifestyle approach to US energy use and the related CO2 emissions. Energy Policy 33: 197–208.

[26] Kok R, Benders R, Moll H (2006) Measuring the environmental load of household consumption using some methods on input-output energy analysis: a comparison of methods and a discussion of results. Energy Policy 34: 2744–2761.

[27] Wright LA, Kemp S, Williams I (2011) ‘Carbon footprinting’: towards a universally accepted definition. Carbon Management 2: 61–72.

[28] Tian GJ, Jiang J, Yang ZF, Zhang YQ (2011) The urban growth, size distribution and spatio-temporal dynamic pattern of the Yangtze River Delta megalopolitan region, China. Ecological Modelling 222: 865–878.

[29] Jin RL, Tian LX, Qian JL, Liu YT (2010) The dynamic evolutionary analysis on carbon emissions in Yangtze Delta. International Journal of Nonlinear Science 10: 259–263.

[30] Zhai SY, Wang Z (2013) Modeling relationship among carbon emission, energy consumption and economic growth by ARDL in the Yangtze River Delta. Resource and Environment in the Yangtze Basin 22: 94–103 (in Chinese).

[31] Fan JL, Liao H, Liang QM, Tatano H, Liu CF, et al (2013) Residential carbon emission evolutions in urban-rural divided China: An end-use and behavior analysis. Applied Energy 101: 323–332.

[32] Gu ZH, Sun Q, Wennersten R (2013) Impact of urban residences on energy consumption and carbon emissions: An investigation in Nanjing, China. Sustainable Cities and Society 7: 52–61.

[33] Intergovernmental Panel on Climate Change (IPCC) (2006) Intergovernmental Panel on Climate Change Guidelines for National Greenhouse Gas Inventories, 2006. Available: http://www.ipcc-nggip.iges.or.jp/public/2006gl/index.html. Accessed 10 July 2013.

[34] Editorial Committee of Annual Report on Development of Small and Medium-Sized Cities in China (2010) Annual Report on Development of Small and Medium-Sized Cities in China 2010: A Green Development Route of Small and Medium-sized Cities in China. Beijing: Social Sciences Academic Press (in Chinese).

[35] Xinhuanet (2014) China's urbanization plan 2014–2020 (in Chinese).Available: http://news.xinhuanet.com/fortune/2014-03/16/c_119791251.htm. Accessed 16 March 2014.

[36] Social Development Squad of the Ministry of Science and Technology, the 21st Agenda Government Center (2007) Handbook of Energy Saving and Emission Reducing. Beijing: Social Sciences Archive Press (in Chinese).

[37] Wang HH, Zhang RR, Bi J (2011) Carbon accounting for Chinese cities-A case of Wuxi City. China Environmental Science, 31: 1029–1038 (in Chinese).

[38] Zhang Q, Tao XM, Yang P (2012) Research on carbon emissions from metropolis urban passenger transport and countermeasures. China Population, Resources and Environment 22: 35–42 (in Chinese).

[39] Xie HY, Wang XX, Yang MZ, Jin CQ, Zhong SJ, et al (2011) The carbon emission analysis of Shenzhen Metro. Acta Ecologica Sinica 31: 3551–3558 (in Chinese).

[40] Zhang T, Le Y, Huang YL, Zhang CX (2012) Carbon accounting and analysis for urban garbage disposal in China: A case of Suzhou city. Environmental Pollution & Control 34: 102–110 (in Chinese).

[41] Boussauw K, Witlox F (2009) Introducing a commute-energy performance index for Flanders. Transportation Research Part A 43: 580–591.

[42] Duffy A, Crawford R (2013) The effects of physical activity on greenhouse gas emissions for common transport modes in European countries. Transportation Research Part D 19: 13–19.

[43] National Development and Reform Commission (2010) Regional Plan in the Yangtze River Delta (2009–2020). Available:http://www.china.com.cn/policy/txt/2010-06/22/content_20320273.htm. Accessed 10 July 2013.

[44] Lü L, Fang X (2010) Analysis on the impacts of private car license-plate auction policy in Shanghai, China. Science & Technology Information 27: 253–254 (in Chinese).

[45] Xinhuanet (2013) China to ease one-child policy. Retrieved 15 Nov. 2013. Available at: http://news.xinhuanet.com/english/china/2013-11/15/c_132891920.htm.

[46] International Energy Agency (2013) CO_2_ Emissions from Fuel Combustion 2013—Highlights. Available:http://www.iea.org/publications/freepublications/publication/CO2EmissionsFromFuelCombustionHighlights2013.pdf. Accessed 29 April 2014.

[47] National People's Congress (2013) A report on urbanization development. Available at: http://www.npc.gov.cn/npc/xinwen/jdgz/bgjy/2013-06/27/content_1798658.htm. Accessed 26 June 2013.

